# Aberrant Assembly Complexes of the Reaction Center Light-harvesting 1 PufX (RC-LH1-PufX) Core Complex of *Rhodobacter sphaeroides* Imaged by Atomic Force Microscopy*

**DOI:** 10.1074/jbc.M114.596585

**Published:** 2014-09-05

**Authors:** John D. Olsen, Peter G. Adams, Philip J. Jackson, Mark J. Dickman, Pu Qian, C. Neil Hunter

**Affiliations:** From the ‡ Department of Molecular Biology and Biotechnology, University of Sheffield, Sheffield, S10 2TN, United Kingdom and; the § Department of Chemical and Biological Engineering, ChELSI Institute, University of Sheffield, Sheffield, S1 3JD, United Kingdom

**Keywords:** Atomic Force Microscopy (AFM), Bacteria, Membrane Biogenesis, Membrane Protein, Photosynthesis, Light Harvesting, Reaction Center

## Abstract

In the purple phototrophic bacterium *Rhodobacter sphaeroides*, many protein complexes congregate within the membrane to form operational photosynthetic units consisting of arrays of light-harvesting LH2 complexes and monomeric and dimeric reaction center (RC)-light-harvesting 1 (LH1)-PufX “core” complexes. Each half of a dimer complex consists of a RC surrounded by 14 LH1 αβ subunits, with two bacteriochlorophylls (Bchls) sandwiched between each αβ pair of transmembrane helices. We used atomic force microscopy (AFM) to investigate the assembly of single molecules of the RC-LH1-PufX complex using membranes prepared from LH2-minus mutants. When the RC and PufX components were also absent, AFM revealed a series of LH1 variants where the repeating α_1_β_1_(Bchl)_2_ units had formed rings of variable size, ellipses, and spirals and also arcs that could be assembly products. The spiral complexes occur when the LH1 ring has failed to close, and short arcs are suggestive of prematurely terminated LH1 complex assembly. In the absence of RCs, we occasionally observed captive proteins enclosed by the LH1 ring. When production of LH1 units was restricted by lowering the relative levels of the cognate *pufBA* transcript, we imaged a mixture of complete RC-LH1 core complexes, empty LH1 rings, and isolated RCs, leading us to conclude that once a RC associates with the first α_1_β_1_(Bchl)_2_ subunit, cooperative associations between subsequent subunits and the RC tend to drive LH1 ring assembly to completion.

## Teaser

**Background:** Photosynthesis relies upon connected pigment protein complexes to capture, transfer, and utilize light energy.

**Results:** Aberrant forms of light-harvesting 1 (LH1) complex occur without the reaction center (RC); complete RC-LH1 complexes and isolated RCs were found when LH1 levels were reduced.

**Conclusion:** The subunits of the RC-LH1 complex of *Rhodobacter sphaeroides* are assembled cooperatively.

**Significance:** This represents a paradigm for assembly of other purple bacterial photosystems.

## Introduction

To grow photosynthetically the purple bacterium *Rhodobacter sphaeroides* forms a highly invaginated intracytoplasmic membrane (ICM)^3^ where the components of the photosystem reside (1). Atomic force microscopy (AFM) studies of bacterial photosynthetic membranes have revealed the organization of photosynthetic complexes in ICM from *R. sphaeroides*; large areas of the peripheral light-harvesting LH2 complex are connected to reaction center (RC)-light-harvesting 1 (LH1)-PufX core complexes, thus providing an efficient light-gathering and energy transmission network of bacteriochlorophylls (Bchls) (2, 3). Similar networks have been observed in other purple bacteria but with either monomeric core complexes mixed with the LH2 complex (4–6) or arrays consisting of only monomeric core complexes as in *Blastochloris viridis* (Ref. 7; see Refs. 8 and 9 for reviews).

To our knowledge, the biogenesis of membrane protein complexes has not been investigated using AFM, despite the advantages of this approach in terms of visualizing single molecules in their native membrane environment, under liquid. More specifically, the assembly of the RC-LH1-PufX complex has not been investigated in detail. The 521-kDa RC-LH1-PufX dimer comprises 64 polypeptide components, 64 Bchl cofactors, and 58 carotenoids (10, 11), and it therefore presents a challenging target for assembly studies. It is known that the RC-H subunit is the first component that appears when highly aerated, almost unpigmented cells, are switched to the low aeration conditions that promote photosystem assembly (12), an observation consistent with earlier work (13), and that the PufX polypeptide is incorporated before the LH1 α and β polypeptides (12). A modular assembly mechanism was proposed for the LH1 complex encircling the RC (12, 14), in which α_1_β_1_(Bchl)_2_ units are progressively added until the final stoichiometry of α_28_β_28_(Bchl)_56_ is reached in the case of the dimeric RC-LH1-PufX complex (10).

To accumulate sufficient levels of assembly products for biochemical and structural analyses, it is sometimes necessary to perturb the latter stages of photosystem biogenesis. This strategy has been widely used to dissect the process of the Photosystem II assembly in the cyanobacterium *Synechocystis* 6803. Mutants lacking various subunits of Photosystem II accumulate distinct transient assembly intermediates (15), which allowed the identification of protein factors involved in Photosystem II biogenesis (16, 17). In this work, we simplified the analysis of AFM topographs by deleting the *pucBA* genes encoding LH2 polypeptides and the *pufX* gene encoding the PufX component of the RC-LH1-PufX dimer, leaving a monomeric RC-LH1 as the sole remaining photosystem complex. The role of PufX in the assembly of the core complex will be addressed in future work. We also limited the production of LH1 subunits relative to RC polypeptides by removing a stem-loop structure between *pufA* and *pufL* (12) and analyzed the effects of depleting LH1 levels. Finally, we analyzed the further consequences of deleting genes encoding the RC L and M polypeptides, which leaves only the “empty” LH1 ring.

A previous AFM study of LH1 complexes solubilized in detergent and then reconstituted into two-dimensional sheets had revealed a variety of distorted LH1 complexes, as well as incomplete rings, spirals, and C-shaped arcs (18). To exclude the possibility of preparation-induced artifacts, we developed a detergent-free membrane isolation procedure. AFM imaging of these membranes show that the combinations of mutations used have disrupted the normal developmental cues that guide the assembly and correct sizing of LH1; in the case of membranes isolated from the LH1-only mutant, we observed variable ring sizes, rings distorted to either elliptical or more extreme morphologies, short arcs, incomplete rings, and also spirals where the ring has failed to close. Reducing the levels of α_1_β_1_(Bchl)_2_ subunits resulted in a subpopulation of isolated RCs that had no evidence of even a partial LH1 complex associated with them, whereas the rest of the RC-LH1 complexes had a complete ring. These single molecule observations reveal complexities of photosystem assembly that would not be detected in ensembles by bulk electrophoretic or spectroscopic techniques. Our data indicate that the assembly of core complexes is driven largely by cooperative associations between α_1_β_1_(Bchl)_2_ subunits around the RC template. We propose a mechanism for formation of α_1_β_1_(Bchl)_2_ subunits and a sequence for their assembly round the RC.

## EXPERIMENTAL PROCEDURES

### 

#### 

##### Strains and Standard Cell Culture

The following LH2-minus mutants of *R. sphaeroides* were used: DD13(pRKEK1), which produces only the LH1 complex, and DD13(pRKEHWT), which produces a mixture of monomeric and dimeric core complexes (19), DD13(pRKEH10X^−^) and DD13(pRKΔTΔX) (20); both make monomeric PufX-minus RC-LH1 core complexes, with the latter mutant lacking the stem-loop structure between *pufB* and *pufL* that ensures a normal LH1:RC stoichiometry. Each of these mutants uses the DD13 background (19), in which both the *pucBA1* and *pufBALMX*, encoding polypeptides of the LH2 and RC-LH1-PufX core complexes, respectively, are deleted. Conjugative transfer of the pRK plasmid variants into DD13 confers the LH1-only, PufX-plus RC-LH1, and PufX-minus RC-LH1 core phenotypes.

All strains were grown in M22+ medium supplemented with casamino acids and vitamins. Semi-aerobic cultures of the LH1-only strain DD13(pRKEK1) and the PufX-minus strain DD13(pRKEH10X^−^) were grown in the dark at 30 °C, using a shaking incubator at 140 rpm, in conical flasks at 75% capacity. DD13(pRKEHWT) and DD13(pRKΔTΔX) were grown phototrophically in 1-liter Roux bottles, presparged with N_2_, at room temperature with constant stirring at a light intensity of ∼500 μmol m^−2^ s^−1^.

##### Preparation of Intracytoplasmic Membranes

ICM and membrane patches were prepared from DD13(pRKEK1) and DD13(pRKEH10X^−^) by broadly the same methodology used by Olsen *et al.* (21). Briefly, cells were disrupted in a French press, and the membranes were separated on a discontinuous 15/40% w/w sucrose density gradient. Five optical density units at 875 nm of these membranes were then layered onto a 30/34/37/40/50% w/w sucrose discontinuous gradient and centrifuged in a Beckman SW41 rotor for 1 h at 40,000 rpm at 4 °C, and the resultant bands were collected. These detergent-free membranes were stored at −20 °C until used for imaging. The detergent-treated DD13(pRKEK1) and DD13(pRKEH10X^−^) membranes were prepared as above, except that they were incubated for 30 min at room temperature with β-dodecylmaltoglucoside (β-DDM) (Glycon Biochemistry, GmbH Biotechnology, Germany) at a final concentration of between 0.1 and 0.2 mm (critical micelle concentration, ∼0.34 mm) and separated on a discontinuous 20/30/40/50% w/w, 10 mm HEPES, pH 7.5, 10 mm EDTA, 0.1/0.2 mm β-DDM sucrose density gradient in a Beckman SW41 rotor for 4 h at 40,000 rpm at 4 °C. The DD13(pRKΔTΔX) membrane preparation was incubated with β-DDM as above but then centrifuged through a 20/30/40/50% w/w discontinuous sucrose gradient containing 0.5 mm *n*-tetradecyl-β-d-maltopyranoside (Anatrace; Affymetrix). The membrane bands collected were diluted with 20 mm HEPES pH 7.5 buffer, between 10- and 20-fold prior to adsorption onto mica substrates (Agar Scientific) and allowed to adsorb for at least 60 min at room temperature.

##### Atomic Force Microscopy

Standard Olympus TR800PSA SiN probes (Atomic Force GmbH, Mannheim, Germany), with a spring constant of 0.15 N/m and Si/SiN SNL probes (Bruker AFM Probes) with a spring constant of 0.12 N/m were used in a standard tapping mode liquid cell at operating frequencies of ∼9 KHz, using a Nanoscope IV AFM and E scanner and a Nanoscope V Multimode 8 with E scanner (Bruker, formerly VEECO). Peak force tapping mode imaging was conducted using SNL probes, 0.24 N/m spring constant, 2-KHz operating frequency in a Nanoscope V Multimode 8 with E scanner. The images were recorded at scan frequencies of 0.5–1.0 Hz and subsequently analyzed as described below.

Topographs were flattened, and three-dimensional representations were generated using Bruker Nanoscope Analysis software (v1.4). Statistical analysis of high resolution topographs was performed using the same software to measure peak to peak of height profiles across individual molecules in line with the scan and orthogonal to it. Only clearly defined complexes were examined to produce the most accurate analysis. For the LH1 diameter measurements, the distance between the maximal protrusions of each side of the LH1 ring was taken (*i.e.* diameter corresponds to a “middle” diameter, in between the inner and outer limits of the ring structure). To establish the presence of protein *versus* lipid in low resolution images, all heights were measured from the mica substrate.

##### Quantitative Proteomic Analysis of Developing Photosynthetic Membranes

Total membrane proteins (30 μg by Bradford assay) from a pigmented membrane fraction previously identified as developing, immature membranes (1) were separated by SDS-PAGE (NuPAGE 12%, 1 mm; Invitrogen). After staining with colloidal Coomassie Blue, the gel was cut into 30 slices that were then incubated with trypsin (22). The extracted tryptic peptides were analyzed by nanoLC-MS/MS and identified by database searching (23). Peptides mapping to core complex subunits PufL, PufM, and PufA were quantified by comparison with the ion intensities of 3 pmol of peptide standards derived from the tryptic digestion of an artificial protein containing the relevant proteotypic peptide sequences (11).

## RESULTS

### 

#### 

##### Aberrant Assembly of the LH1 Ring in the Absence of PufX and RC Complexes: the Formation of Variable Ring Sizes and Morphologies and the Presence of Short Arcs and Linear LH1 Assemblies

**FIGURE 1.**
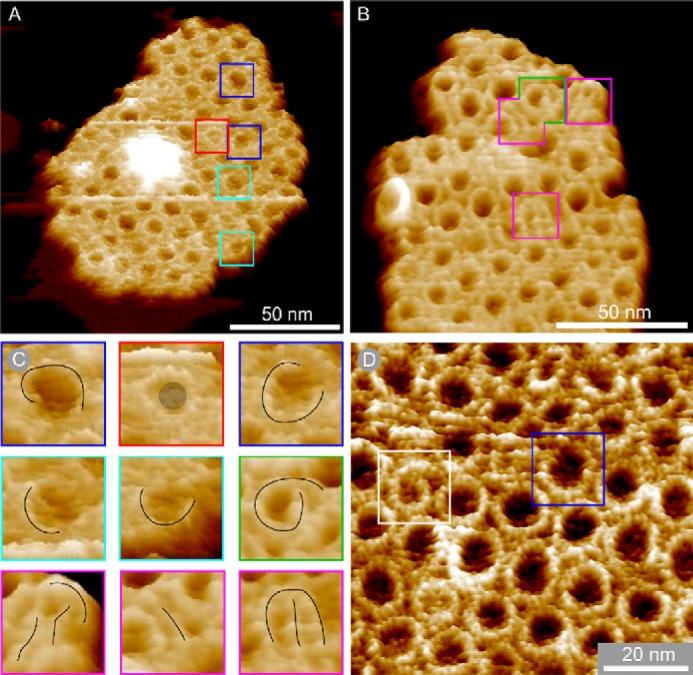
**LH1-only membrane patches in three-dimensional representation showing aberrant complexes.** *A* and *B*, membrane patches produced with no detergent treatment. *C*, gallery of close-ups of boxed complexes in *A* and *B* with *black line* overlays showing the shape of the aberrant complexes and a *gray circle* denoting a captured protein in the complex marked with a *red box. D*, membrane patch prepared using sub-CMC levels of β-DDM. The complex marked with a *white box* is a spiral, and the complex marked with a *blue box* is an open ring. The data in *B* and *D* have been treated with a low pass filter to reduce noise.

Peak force tapping AFM was used to image a representative range of membrane patches from the LH1-only mutant DD13(pRKEK1) at a resolution high enough to visualize the size and shape of the LH1 ring (Fig. 1). We note the lack of any protein within the majority of the LH1 complexes imaged, which indicates that the RC H-subunit does not appear to associate with the LH1 complex in the absence of the RC L and M subunits (Fig. 1, *A* and *B*).

The dimensions of the LH1 complexes were extracted from AFM topographs in orthogonal orientations to obtain a realistic assessment of the size and flexibility of the LH1 ring both with and without the RC. The data, summarized in Table 1, show that the LH1-only rings are smaller on average (width, 9.9 ± 0.7 nm; height, 10.6 ± 0.9 nm; S.D., *n* = 21), in comparison with monomeric RC-LH1 core complexes (width, 11.2 ± 0.6 nm; height, 11.3 ± 0.6 nm; S.D., *n* = 9).

**TABLE 1:** **The average diameter of the LH1 ring, with and without the RC when prepared with and without detergents** The average diameter and standard deviation of the LH1 ring, with and without the RC, in line with the scan direction (width) and orthogonal to the scan direction (height). The diameter was measured between the highest points of the ring, *i.e.* the average ring diameter. There is no significant difference in the dimensions regardless of whether detergent has been used in the preparation of the membrane samples. The smaller number of diameters measured of core complexes represents the difficulty in obtaining AFM tips that are sufficiently sharp to resolve the ring when it is in such close proximity to the protruding H-subunit.

	Width	Height	Rings measured (*n*)
	*nm*	*nm*	
LH1-only with detergent	10.1 ± 0.9	10.0 ± 1.6	61
RC-LH1 with detergent	11.2 ± 0.5	11.5 ± 0.6	12
LH1-only no detergent	9.9 ± 0.7	10.6 ± 0.9	21
RC-LH1 no detergent	11.2 ± 0.6	11.3 ± 0.6	9

**FIGURE 2.**
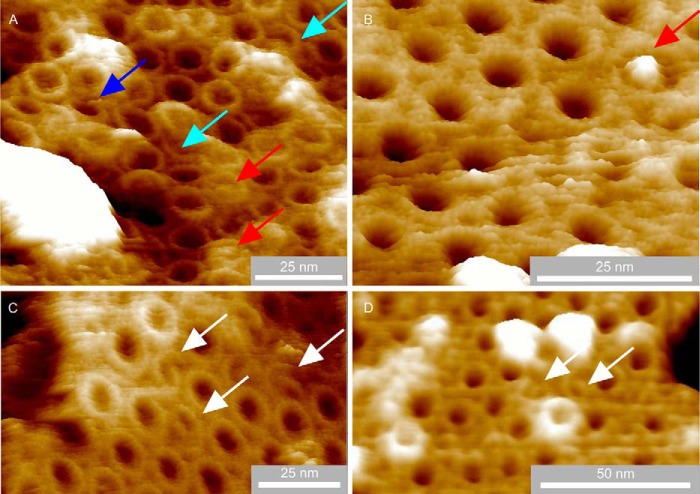
**LH1-only membrane patches prepared using sub-CMC concentration β-DDM detergent in three-dimensional representation showing aberrant complexes.** *A*, a piece of membrane showing a variety of aberrant LH1 complexes including an incomplete ring (*blue arrow*), arcs (*cyan arrows*), and captive proteins (*red arrows*). *B*, a particularly well resolved captive protein with high topology (*red arrow*). *C*, examples of spiral complexes (*white arrows*). *D*, further examples of spiral complexes (*white arrows*). The image data have been treated with a low pass filter to reduce noise.

In patches imaged to higher resolution (Fig. 1, *A–D*), we identified several aberrant forms of LH1, including incomplete rings (*blue boxes*), spirals (*green* and *white boxes*), arcs (*cyan boxes*), and near linear features (*magenta boxes*). In *A* and *B*, the membranes were prepared without using detergent, whereas the membrane piece in *D* had been prepared using sub-CMC levels of β-DDM, as detailed under “Experimental Procedures”. The appearance of many deformed LH1 complexes in both sets of membrane patches indicates that the method of membrane preparation has no influence on the morphology of the complexes (Fig. 1, *A–D*, *colored boxes*). In the cases marked by the *green* and *white boxes*, a gently curved row of α_1_β_1_(Bchl)_2_ subunits is seen (Fig. 1, *B*, and *D*, respectively), making a continuous structure with the spirals. Short, almost linear structures (Fig. 1*B*, *magenta boxes*) can be seen in the enlargement in Fig. 1*C*, which, like the incomplete rings, could be “stalled” LH1 α_1_β_1_(Bchl)_2_ oligomers that failed to polymerize sufficiently to form a closed complex. More examples of β-DDM detergent prepared membranes containing aberrant LH1 complexes with distorted rings, incomplete rings, arcs, and spirals are shown in Fig. 2. In total, we observed 37 deformed LH1 complexes in both sets of membrane patches representing 304 LH1 complexes in total, whereas no such structures were observed in membranes from wild-type cells.

##### Aberrant Assembly of the LH1 Ring in the Absence of PufX and RC Complexes: Occasional Capture of Unknown Proteins within LH1 Rings

**FIGURE 3.**
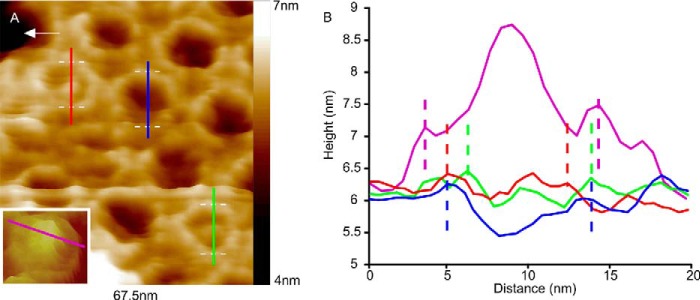
**Putative captive proteins enclosed by LH1 rings.** *A*, close-up of the LH1-only membrane in Fig. 1*A*; sections were taken across three LH1 complexes: one across an empty LH1 ring (*blue line*) and two across rings with a central density (*red* and *green sections*). The *inset* shows a topograph of a monomeric RC-LH1 (no PufX) complex for comparison. *B*, sections taken across the LH1 complexes shown in *A*; the *magenta section* was taken across an RC-LH1 complex from a separate sample of membranes containing monomeric RC-LH1 complexes. The *vertical dashed lines* indicate the highest points of the LH1 ring. The image data have been treated with a low pass filter to reduce noise; the section data have not been filtered.

An enlargement of the LH1-only membrane AFM data from Fig. 1*A* highlights three LH1 complexes, with corresponding colored height profiles, that show two encircled proteins compared with an empty LH1 ring (Fig. 3*A*). The *inset* is the topograph of an RC-LH1 complex showing the prominent topology of the RC-H subunit, which clearly protrudes much more than the captive proteins (Fig. 3*B*, *magenta section*). The *vertical dashed lines* on the sections of Fig. 3*B* denote the highest points of the LH1 ring, which highlights the relatively low topology of the captive proteins, shown by the *red* and *green sections*, in relation to a RC-H subunit (*magenta line*). The greater diameter of the LH1 complex surrounding the RC is also clear in this section. In contrast, when no protein is enclosed by LH1, the AFM probe penetrates more deeply into the LH1 interior (*blue section*), which is nevertheless 5.4 nm above the mica substrate, suggesting that the interior of the complex is filled with lipid. Of 181 clearly defined complexes, only 3 contained unambiguous central density, indicating the presence of a captive protein. The results for LH1-only membranes prepared using mild detergents (Fig. 2, *A* and *B*) mirror those from entirely detergent-free preparations in Figs. 1 and 3, where of 123 complexes three contained central density consistent with captive proteins and demonstrate that oligomerization of α_1_β_1_(Bchl)_2_ subunits can occasionally capture foreign proteins when the RC complex they normally encircle is absent.

##### Aberrant Membrane Architecture in the Absence of PufX and LH2 Complexes: Identification of LH1-only Complexes

**FIGURE 4.**
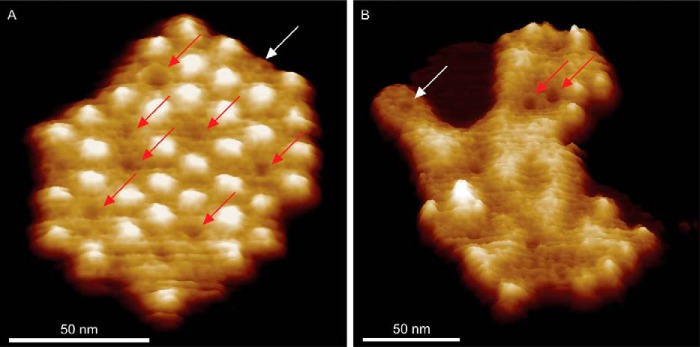
**Three-dimensional representations of RC-LH1 membrane patches showing empty LH1 rings.** *A*, empty LH1 rings (*red arrows*) within an RC-LH1 array; one core complex (*white arrow*) has lost its H-subunit probably as a result of the scanning by the AFM tip. *B*, a less well defined membrane patch where two prominent empty LH1 rings can be seen beside three core complexes, as well as some nanodissected cores, of which the best example is indicated by the *white arrow*. The data have been treated with a low pass filter to reduce noise.

Fig. 4*A* shows an ICM-derived patch prepared without detergent from an LH1^+^ RC^+^ PufX^−^ LH2^−^ mutant, which assembles only monomeric RC-LH1 complexes. The AFM topographs show that the membrane consists mainly of well ordered RC-LH1 monomers; however, the lattice is interrupted in places (*red arrows*) by a surprisingly large number of empty LH1 rings of varying size. Inspection of the four scans prior to this image rules out the possibility of repeated scanning inducing the removal of the RC because there is no sign of an intact RC at the positions of the empty LH1 rings (data not shown), which would be expected if AFM-induced damage had occurred. There is only one example of an LH1 ring with partial central density (*white arrow*), which is expected to represent RC-L and RC-M. A similar situation occurs in the membrane patch in Fig. 4*B* where two empty LH1 rings can be seen adjacent to intact RC-LH1 complexes; the two prior scans show no evidence of the RC within either of these LH1 rings. Elsewhere in the patch, a number of RC-LH1 complexes lacking a H subunit occur (example indicated with *white arrow*).

The presence of empty LH1 rings in this PufX^−^ LH2^−^ mutant implies an excess of LH1 polypeptides over RC subunits. To address this issue, we used quantitative proteomic analysis to determine the ratio of LH1 α to RC polypeptides in developing photosynthetic membranes of *R. sphaeroides*. Our data show that the mean ratio of LH1 α to the RC-L and -M subunits in the membrane precursor fraction is 29:1. To put this in perspective, a ratio of 14:1 is necessary to form an RC-LH1-PufX dimer complex, as shown by a recent structural study (11). These mass spectrometry data clearly show that the biogenesis centers in *R. sphaeroides* produce a molar excess of LH1 polypeptides over the RC components.

**FIGURE 5.**
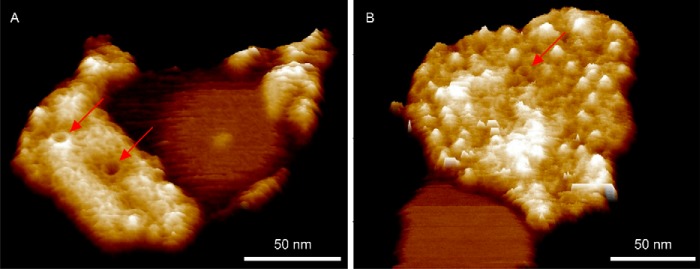
**Three-dimensional representations of RC-LH1 membrane patches prepared using a sub-CMC concentration β-DDM detergent, showing empty LH1 rings.** *A*, a membrane patch of monomeric RC-LH1 complexes where most of the H-subunits are missing revealing the L- and M-subunits; two clear empty LH1 rings are denoted by the *red arrows. B*, a less well resolved patch of RC-LH1 complexes showing a small, ∼8-nm-diameter, empty LH1 ring (*red arrow*).

These data support the proposal that the empty LH1 complexes are the products of aberrant assembly. Empty LH1 complexes have also been observed in RC-LH1-only membrane patches prepared using β-DDM (Fig. 5).

##### Consequences of Limited α_1_β_1_(Bchl)_2_ Subunit Production upon Assembly of RC-LH1 Core Complexes

**FIGURE 6.**
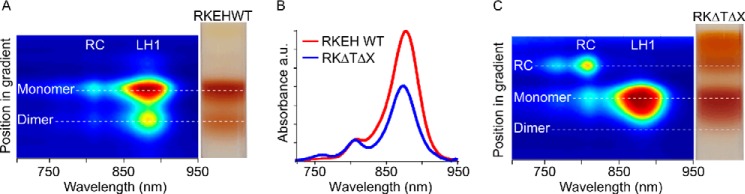
**Analysis of the absorption and sedimentation behavior of solubilized complexes from membranes prepared from the RC-LH1 and LH1-limited mutants.** *A*, two-dimensional plot of room temperature NIR absorbance spectra of complexes solubilized from the LH1-limted DD13(pRKEHWT) mutant, taken at 2-mm intervals through a discontinuous sucrose density gradient, where *blue* indicates no absorption and the spectrum through to *red* indicates increasing levels of absorption, aligned with a photograph of the gradient. *B*, room temperature NIR absorption spectra of DD13(pRKEHWT) and DD13(pRKΔTΔX) membranes normalized to the RC 805-nm band. *C*, two-dimensional plot of room temperature NIR absorption spectra of complexes solubilized from the LH1-limited mutant, DD13(pRKΔTΔX), aligned with a photograph of the gradient.

To investigate the effects of restricting the number of LH1 subunits on assembly of core complexes, we used the “small core” mutant DD13(pRKΔTΔX) (12). Here, deletion of a stem-loop between *pufA* and *L* genes lowers the levels of the LH1 *pufBA* transcript with respect to the RC-LH1 transcript encoded by *pufBALM*, with the effect that the absorbance of LH1 is lowered by ∼43% (Fig. 6*B*). To verify the presence of isolated, LH1-free RCs in these membranes we performed a biochemical analysis using rate-zone centrifugation of detergent-solubilized membranes (Fig. 6, *A* and *C*), which separates the complexes into an upper monomeric band and a lower dimeric core complex band. The small core mutant DD13(pRKΔTΔX) (Fig. 6*C*) consists largely of monomer core complexes with an LH1:RC ratio, approximated by the 875:805 absorbance ratio of 3.6:1, compared with 6.2:1 in the WT control. The room temperature absorption spectrum of the well defined band above the monomer band indicates that it is composed of RCs with no accompanying LH1 complex. The *yellow* and *orange bands* at the top of both gradients are probably free carotenoids. These data are consistent with the presence of isolated RCs in the membrane of the LH1-limited DD13(pRKΔTΔX) mutant.

**FIGURE 7.**
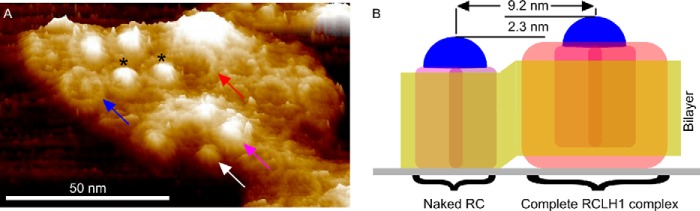
**AFM analysis of a membrane patch prepared from the LH1-limited mutant DD13(pRKΔTΔX).** *A*, three-dimensional representation of a membrane patch exhibiting examples of an intact RC-LH1 complex (*magenta arrow*), an isolated RC (*white arrow*), an RC-LH1 complex missing the H-subunit (*blue arrow*), and an empty LH1 ring (*red arrow*). *B*, schematic diagram to illustrate the expected height difference of an isolated RC, which would rest directly upon the mica substrate, in comparison with an intact RC-LH1 complex where the C-terminal region of the LH1 ring rests on the mica, holding the RC above it.

Membranes were prepared from photosynthetically grown cells of the LH1-limited mutant and analyzed by AFM. A high resolution topograph of a membrane patch (Fig. 7*A*) revealed that not only were complete RC-LH1 complexes present (*magenta arrow*), but also an isolated RC (*white arrow*) and empty LH1 rings (*red arrow*) could be imaged. The *blue arrow* shows a core complex with the RC-H subunit missing. Isolated RCs were observed in several AFM images and identified by their height profile in comparison with nearby core complexes, confirming the biochemical analysis shown in Fig. 6.

In the topograph there is a 2.3-nm height difference between the top of the RC-H subunit within an intact RC-LH1 complex (*magenta arrow*) and the RC-H of the isolated RC (*white arrow*). This height difference between the RC-only and the RC-LH1 complexes in the membrane is apparent because the C-terminal extrinsic region of the LH1α polypeptides in the ring normally raises the RC and the membrane bilayer off the mica surface (24). The visual absence of an LH1 ring around the RC marked with a *white arrow* is supported by the 9.2 nm center to center distance to the RC marked with a *magenta arrow*, whereas the equivalent distance between two well resolved RC-LH1 complexes (marked with *asterisks*) is 13.3 nm. Fig. 7*B* is a diagrammatic representation of the height data for the two adjacent complexes in the topograph, an isolated RC (*white arrow*) that clearly has no LH1 encircling it and a complete RC-LH1 complex (*magenta arrow*). Taken together with the biochemical analysis in Fig. 6, it is likely that the limited number of LH1 α_1_β_1_(Bchl)_2_ subunits are not shared equally among the RCs present; instead the assembly process favors completion of ring formation at the expense of some RCs, which are left with no LH1 complex.

## DISCUSSION

This work reports the first AFM study of RC-LH1 assembly, using membranes of *R. sphaeroides* containing only RC-LH1 or LH1 complexes prepared without the use of detergents, demonstrating that the various *in situ* aberrant LH1 forms are the product of perturbed assembly. Similar aberrant forms were previously observed in LH1 complexes purified from LH1-only membranes in 1% β-octyl glucoside and then reconstituted into two-dimensional sheets by gradual removal of the detergent (18). In the present study, topographs of native membrane patches, prepared without detergents, reveal new insights into the factors that control the size and shape of LH1 complexes and drive the encirclement of the RC to completion. Single complexes within LH1-only and RC-LH1 membranes were imaged at a resolution sufficient for a comparison of the dimensions of the complexes; the LH1-only complex is smaller on average than the RC-LH1 monomeric core complex. Moreover, the variation in LH1 size, even in the presence of the RC (Table 1), is an indicator of a very simple assembly system without rigid quality control.

**FIGURE 8.**
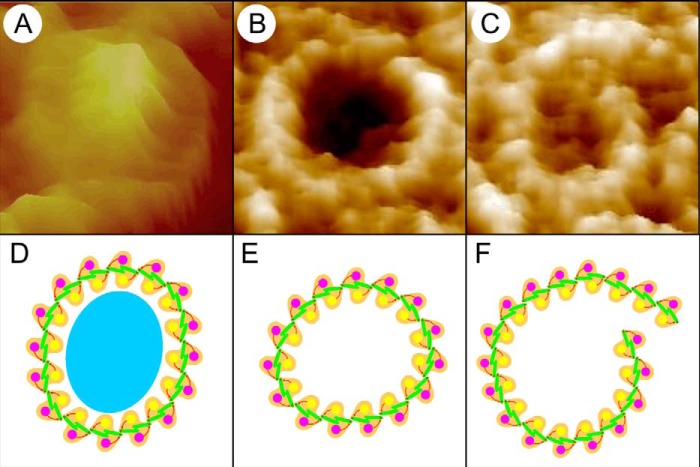
**Three individual complexes and cartoons of their assigned composition.** *A*, an RC-LH1 core complex. *B*, an LH1 complex. *C*, an aberrant spiral LH1 complex. *D*, cartoon of an RC, cyan oval, surrounded by 16 α_1_β_1_(Bchl)_2_ subunits. *E*, a slightly distorted ring of 15 α_1_β_1_(Bchl)_2_ subunits. *F*, a spiral composed of 18 α_1_β_1_(Bchl)_2_ subunits.

Fig. 8 (*A–C*) shows three LH1 complexes imaged at high resolution by AFM, together with schematic approximations of α_1_β_1_(Bchl)_2_ subunits showing how they might be arranged in each complex (Fig. 8, *D–F*). The complete LH1 ring surrounding the RC (Fig. 8*D*, *cyan oval*) has been assigned as 16 α_1_β_1_(Bchl)_2_ subunits arranged in a roughly circular ring, in accordance with existing cryo-electron microscopy data showing that 16 LH1 subunits enclose a single RC (25). The presence of complete LH1 rings surrounding the *R. sphaeroides* RC in the same PufX^−^ mutant have been characterized by electron microscopy (26, 27) and in a similar PufX^−^ LH2^+^ mutant by AFM (28). The empty LH1 ring (Fig. 8*B*) is depicted as 15 α_1_β_1_(Bchl)_2_ subunits (Fig. 8*E*), consistent with the slightly smaller diameter seen for such rings in AFM topographs (Fig. 3 and Table 1), whereas the large spiral (Fig. 8*C*) has been tentatively assigned as 18 α_1_β_1_(Bchl)_2_ subunits (Fig. 8*F*). As the predominant morphology of LH1-only complexes is a closed circular structure, it is clear that the α_1_β_1_(Bchl)_2_ subunits naturally form curved arrays that tend to close once 15 or 16 subunits are joined together. Cryo-electron microscopy of *Rhodospirillum rubrum* LH1 complexes, reconstituted from α_1_β_1_(Bchl)_2_ units then dialyzed to form two-dimensional crystals, clearly shows 16-fold rings that are spectroscopically identical to the WT LH1 (29). Loach and co-workers (30–33) have also shown in many studies that *R. sphaeroides* LH1 α_1_β_1_(Bchl)_2_ (B820) units can self-assemble *in vitro* to form complete LH1 α_16_β_16_(Bchl)_32_ rings. In other work, they performed a series of reconstitution trials using LH1 polypeptides from *R. sphaeroides* and the RC from *R. rubrum* to form a spectroscopic photoreceptor complex (34); from this result they proposed that there must be sites of recognition between the homologous LH1 and RC complexes that encourage a RC-LH1 complex to form. From these studies, it seems clear that the LH1 complex naturally forms ring-shaped structures even in the absence of a RC template.

Although there is as yet no quantification of the forces involved in LH1 assembly, a recent force spectroscopy study by Liu *et al.* (35) quantified the Δ*G* for unfolding an LH2 α_1_β_1_(Bchl)_3_ subunit as 53 kcal mol^−1^. We might reasonably expect a similar Δ*G* for an LH1 α_1_β_1_(Bchl)_2_ subunit, which suggests that in a typical LH1-only complex, cumulative small intersubunit forces are a significant driving force for ring formation. The presence of a spiral, such as that in Fig. 8*C*, indicates that in the absence of an RC template it only takes a minor perturbation of the nascent LH1, perhaps by crowding pressure of adjacent completed rings, to push one end out of alignment so that more subunits than are necessary are added, thus extending the complex into a spiral.

**FIGURE 9.**
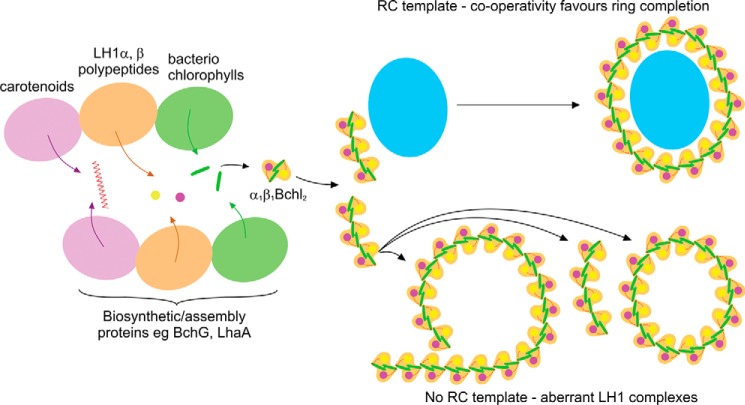
**A schematic representation of the assembly system of *Rhodobacter sphaeroides* light-harvesting complex 1 and RC-LH1 core complexes.** In the absence of LH2 and PufX polypeptides, the pigments and polypeptides that comprise LH1 are still inserted into the membrane and self-assemble into α_1_β_1_(Bchl)_2_ subunits; they associate to form LH1 complexes in the absence of RCs or predominantly complete core complexes in the presence of RCs. Aberrant forms of LH1, *i.e.* varying ring size, arcs, and spirals, occur in the absence of the RC template.

The most striking result of this study is the observation that partial LH1 rings do not occur when RCs are present, providing evidence that the RC clearly acts as a template for the developing LH1 complex. We suggest that, even in the absence of PufX, either a single α_1_β_1_(Bchl)_2_ or a short α_1_β_1_(Bchl)_2_ array associates with the RC (Fig. 9), and thereafter the cooperative forces of self-association between α_1_β_1_(Bchl)_2_ subunits, and likely also with the RC, drive the efficient encirclement to form a completed core complex. This suggestion is consistent with earlier work, which studied the assembly of photosynthetic membranes by switching highly aerated cells with almost no pigment to conditions of low aeration in the dark that induce complex formation (12). Over the 6-h time course of the experiment the RC-H subunit was detected before other components, followed by the PufX polypeptide and then a small level of LH1 α and β polypeptides, which subsequently increases. It was proposed that the earliest stages of assembly involve formation of a RC-PufX-(LH1α_1_β_1_Bchl)_2_ complex, to which α_1_β_1_(Bchl)_2_ units are progressively added, ultimately encircling the RC (12).

The presence of empty LH1 rings in the RC-LH1 PufX^−^ mutants implies an excess of LH1 polypeptides in this membrane. We tested this idea by using quantitative mass spectrometry to measure levels of the LH1 α polypeptide in membrane biogenesis centers of *R. sphaeroides* (1). The data show that in these centers there is a 2-fold excess of LH1 α, relative to the level required to form a RC-LH1-PufX complex. It is possible that rather than having precisely metered delivery of pigments and proteins, the assembly system might be set up to favor an excess of LH polypeptides to “mop up” Bchls that might otherwise be free in the membrane. This mechanism would avoid a potentially damaging situation in a photosynthetic organism where both light and oxygen may be present in sufficient levels to generate oxygen triplet states from unquenched Bchls. Spectroscopic studies have also shown the presence of empty LH1 rings; excitation transfer connectivity calculations (36) revealed that membranes of the mutant R-26 of *R. sphaeroides* were likely to contain significant numbers of empty LH1 rings. This proposal was subsequently supported by the AFM images of R-26 membranes (37). It should also be noted that an empty LH1 complex need not be inactive; it will still act as a light-harvesting unit and as an excitation energy conduit between RC-LH1 complexes.

We had previously removed the stem-loop structure between the *pufA* and *L* genes in the *pufQBALMX* operon to reduce the LH1:RC ratio (12); similar effects can also be achieved by progressive truncation of the C-terminal domain of the LH1α polypeptide (38). Ensemble spectra in these previous studies showed a significant drop in the 875 nm absorbance arising from LH1 complexes relative to the 805-nm absorbance maximum of the monomeric Bchls of the RC. The absorbance spectra in the present work confirm the lowered levels of LH1 (Fig. 6*B*) and appear to suggest a disrupted assembly process in which each of the RCs is only partially enclosed by LH1. However, AFM imaging of membranes to the single molecule level reveals that the scarce LH1 units are not evenly distributed among the RCs; rather, the encircling process, once initiated, appears to proceed to completion. The consequence is a population of fully encircled RCs, *i.e.* “intact” RC-LH1, and also some isolated RCs with no LH1 attached. The appearance of an isolated RC band in rate zonal centrifugation of solubilized membranes (Fig. 6*C*) of this small core mutant, but not in the WT equivalent solubilized membranes, supports this proposal. Core complexes lacking RC-H subunits could be a consequence of enhanced AFM tip induced nano-dissection caused by the absence of PufX, because similar observations have been made for membranes containing RC-LH1 PufX^−^ core complexes of *R. sphaeroides* (28). The three-dimensional crystal structure of the dimeric core complex (11) shows that the PufX N-terminal region (residues 21–30) associates with the H-subunit, which core dimer stabilizes the dimeric form of this complex (39, 40). However, the presence of empty LH1 rings is somewhat surprising in a mutant that produces insufficient α_1_β_1_(Bchl)_2_ subunits to encircle every RC complex and is indicative of a very simple assembly process with little quality control.

The presence of “captive” proteins within the LH1 complexes in the LH1-only mutant highlights the nonspecific nature of the assembly process and demonstrates that in the absence of the reaction center template, LH1 subunits can assemble around another membrane protein of approximately the correct dimensions. Candidates for such proteins include the bacteriochlorophyll synthase BchG, which is predicted to have up to eight transmembrane helices (41), and LhaA, an assembly factor for core complexes, predicted to have 12 transmembrane helices (42). These proteins are expected to be associated with the LH1 assembly process, but not to be detected in association with complete LH1 rings; usually the RC L and M polypeptides would be present and favorable interactions between the α_1_β_1_(Bchl)_2_ subunits and the RC-L, -M, and -H subunits would minimize the chances of LH1 forming around any other membrane protein. At this time, we do not know the actual role of LhaA in the assembly of the core complex, although it has been proposed to be a bacteriochlorophyll delivery export permease (42). When *lhaA* is deleted, the levels of LH1 complex are significantly reduced, 5–10-fold in comparison with the WT, but RC assembly was largely unaffected (43). However, the fact that some core complex does correctly assemble in the absence of LhaA indicates that although this protein greatly enhances assembly, it is not absolutely essential. These data are consistent with the notion that the core complex of *R. sphaeroides* is the product of an assembly process in which cooperative hydrophobic and hydrophilic associations between the various polypeptides heavily bias the system toward formation of complete rings of LH1 around the RC template.

## Footnotes

3 The abbreviations used are:

ICM: intracytoplasmic membrane
AFM: atomic force microscopy
BChl: bacteriochlorophyll
CMC: critical micelle concentration
LH1: light-harvesting 1 complex
LH2: light-harvesting 2 complex
RC: reaction center
RC-LH1: reaction center-light-harvesting 1 complex
β-DDM: β-dodecylmaltoglucoside.

## References

[1] Tucker J. D. Siebert C. A. Escalante M. Adams P. G. Olsen J. D. Otto C. Stokes D. L. Hunter C. N. (2010) Membrane invagination in *Rhodobacter sphaeroides* is initiated at curved regions of the cytoplasmic membrane, then forms both budded and fully detached spherical vesicles. Mol. Microbiol. 76, 833–847 20444085 10.1111/j.1365-2958.2010.07153.x

[2] Bahatyrova S. Frese R. N. Siebert C. A. Olsen J. D. Van Der Werf K. O. Van Grondelle R. Niederman R. A. Bullough P. A. Otto C. Hunter C. N. (2004) The native architecture of a photosynthetic membrane. Nature 430, 1058–1062 15329728 10.1038/nature02823

[3] Frese R. N. Siebert C. A. Niederman R. A. Hunter C. N. Otto C. van Grondelle R. (2004) The long-range organization of a native photosynthetic membrane. Proc. Natl. Acad. Sci. U.S.A. 101, 17994–17999 15601770 10.1073/pnas.0407295102 PMC539794

[4] Scheuring S. Rigaud J. L. Sturgis J. (2004) Variable LH2 stoichiometry and core clustering in native membranes of *Rhodspirillum photometricum*. EMBO J. 23, 4127–4133 15457213 10.1038/sj.emboj.7600429 PMC524393

[5] Gonçalves R. P. Bernadac A. Sturgis J. N. Scheuring S. (2005) Architecture of the native photosynthetic apparatus of *Phaeospirillum molischianum*. J. Struct. Biol. 152, 221–228 16330228 10.1016/j.jsb.2005.10.002

[6] Scheuring S. Gonçalves R. P. Prima V. Sturgis J. N. (2006) The photosynthetic apparatus of *Rhodopseudomonas palustris*: structures and organization. J. Mol. Biol. 358, 83–96 16500674 10.1016/j.jmb.2006.01.085

[7] Scheuring S. Seguin J. Marco S. Lévy D. Robert B. Rigaud J. L. (2003) Nanodissection and high-resolution imaging of the *Rhodopseudomonas viridis* photosynthetic core complex in native membranes by AFM. Proc. Natl. Acad. Sci. U.S.A. 100, 1690–1693 12574504 10.1073/pnas.0437992100 PMC149894

[8] Scheuring S. Lévy D. Rigaud J. L. (2005) Watching the components of photosynthetic bacterial membranes and their in situ organisation by atomic force microscopy. Biochim. Biophys. Acta 1712, 109–127 15919049 10.1016/j.bbamem.2005.04.005

[9] Sturgis J. N. Tucker J. D. Olsen J. D. Hunter C. N. Niederman R. A. (2009) Atomic force microscopy studies of native photosynthetic membranes. Biochemistry 48, 3679–3698 19265434 10.1021/bi900045x

[10] Qian P. Hunter C. N. Bullough P. A. (2005) The 8.5 Å projection structure of the core RC-LH1-PufX dimer of *Rhodobacter sphaeroides*. J. Mol. Biol. 349, 948–960 15907932 10.1016/j.jmb.2005.04.032

[11] Qian P. Papiz M. Z. Jackson P. J. Brindley A. A. Ng I. W. Olsen J. D. Dickman M. J. Bullough P. A. Hunter C. N. (2013) Three-dimensional structure of the *Rhodobacter sphaeroides* RC-LH1-PufX complex: dimerization and quinone channels promoted by PufX. Biochemistry 52, 7575–7585 24131108 10.1021/bi4011946

[12] Pugh R. J. McGlynn P. Jones M. R. Hunter C. N. (1998) The LH1-RC core complex of *Rhodobacter sphaeroides*: interaction between components, time-dependent assembly, and topology of the PufX protein. Biochim. Biophys. Acta 1366, 301–316 9814844 10.1016/s0005-2728(98)00131-5

[13] Chory J. Donohue T. J. Varga A. R. Staehelin L. A. Kaplan S. (1984) Induction of the photosynthetic membranes of *Rhodopseudomonas sphaeroides*: biochemical and morphological studies. J. Bacteriol. 159, 540–554 6611335 10.1128/jb.159.2.540-554.1984 PMC215678

[14] Westerhuis W. H. Sturgis J. N. Ratcliffe E. C. Hunter C. N. Niederman R. A. (2002) Isolation, size estimates, and spectral heterogeneity of an oligomeric series of light-harvesting 1 complexes from *Rhodobacter sphaeroides*. Biochemistry 41, 8698–8707 12093288 10.1021/bi011663b

[15] Komenda J. Reisinger V. Müller B. C. Dobáková M. Granvogl B. Eichacker L. A. (2004) Accumulation of the D2 protein is a key regulatory step for assembly of the photosystem II reaction center complex in *Synechocystis* PCC 6803. J. Biol. Chem. 279, 48620–48629 15347679 10.1074/jbc.M405725200

[16] Dobáková M. Sobotka R. Tichý M. Komenda J. (2009) Psb28 protein is involved in the biogenesis of the photosystem II inner antenna CP47 (PsbB) in the cyanobacterium *Synechocystis* sp. PCC 6803. Plant Physiol. 149, 1076–1086 19036835 10.1104/pp.108.130039 PMC2633855

[17] Komenda J. Knoppová J. Kopečná J. Sobotka R. Halada P. Yu J. Nickelsen J. Boehm M. Nixon P. J. (2012) The Psb27 assembly factor binds to the CP43 complex of photosystem II in the cyanobacterium *Synechocystis* sp. PCC 6803. Plant Physiol. 158, 476–486 22086423 10.1104/pp.111.184184 PMC3252115

[18] Bahatyrova S. Frese R. N. van der Werf K. O. Otto C. Hunter C. N. Olsen J. D. (2004) Flexibility and size heterogeneity of the LH1 light harvesting complex revealed by atomic force microscopy: functional significance for bacterial photosynthesis. J. Biol. Chem. 279, 21327–21333 14993213 10.1074/jbc.M313039200

[19] Jones M. R. Fowler G. J. Gibson L. C. Grief G. G. Olsen J. D. Crielaard W. Hunter C. N. (1992) Mutants of *Rhodobacter sphaeroides* lacking one or more pigment-protein complexes and complementation with reaction-centre, LH1, and LH2 genes. Mol. Microbiol. 6, 1173–1184 1588816 10.1111/j.1365-2958.1992.tb01556.x

[20] McGlynn P. Hunter C. N. Jones M. R. (1994) The *Rhodobacter sphaeroides* PufX protein is not required for photosynthetic competence in the absence of a light harvesting system. FEBS Lett. 349, 349–353 8050595 10.1016/0014-5793(94)00701-2

[21] Olsen J. D. Tucker J. D. Timney J. A. Qian P. Vassilev C. Hunter C. N. (2008) The organization of LH2 complexes in membranes from *Rhodobacter sphaeroides*. J. Biol. Chem. 283, 30772–30779 18723509 10.1074/jbc.M804824200 PMC2662159

[22] Pandey A. Andersen J. S. Mann M. (2000) Use of mass spectrometry to study signalling pathways. Sci. STKE 2000, pl1 11752594 10.1126/stke.2000.37.pl1

[23] Jackson P. J. Lewis H. J. Tucker J. D. Hunter C. N. Dickman M. J. (2012) Quantitative proteomic analysis of intracytoplasmic membrane development in *Rhodobacter sphaeroides*. Mol. Microbiol. 84, 1062–1078 22621241 10.1111/j.1365-2958.2012.08074.x

[24] Fotiadis D. Qian P. Philippsen A. Bullough P. A. Engel A. Hunter C. N. (2004) Structural analysis of the RC-LH1 photosynthetic core complex of *Rhodospirillum rubrum* using atomic force microscopy. J. Biol. Chem. 279, 2063–2068 14578348 10.1074/jbc.M310382200

[25] Jamieson S. J. Wang P. Qian P. Kirkland J. Y. Conroy M. J. Hunter C. N. Bullough P. A. (2002) Projection structure of the photosynthetic reaction centre-antenna complex of *Rhodospirillum rubrum* at 8.5 Å resolution. EMBO J. 21, 3927–3935 12145194 10.1093/emboj/cdf410 PMC125403

[26] Walz T. Jamieson S. J. Bowers C. M. Bullough P. A. Hunter C. N. (1998) Projection structures of three photosynthetic complexes from *Rhodobacter sphaeroides*: LH2 at 6 Å, LH1 and RC-LH1 at 25 Å. J. Mol. Biol. 282, 833–845 9743630 10.1006/jmbi.1998.2050

[27] Siebert C. A. Qian P. Fotiadis D. Engel A. Hunter C. N. Bullough P. A. (2004) Molecular architecture of photosynthetic membranes in *Rhodobacter sphaeroides*: the role of PufX. EMBO J. 23, 690–700 14765115 10.1038/sj.emboj.7600092 PMC381000

[28] Adams P. G. Mothersole D. J. Ng I. W. Olsen J. D. Hunter C. N. (2011) Monomeric RC-LH1 core complexes retard LH2 assembly and intracytoplasmic membrane formation in PufX-minus mutants of *Rhodobacter sphaeroides*. Biochim. Biophys. Acta 1807, 1044–1055 21663730 10.1016/j.bbabio.2011.05.019

[29] Karrasch S. Bullough P. A. Ghosh R. (1995) The 8.5 Å projection map of the light-harvesting complex I from *Rhodospirillum rubrum* reveals a ring composed of 16 subunits. EMBO J. 14, 631–638 7882966 10.1002/j.1460-2075.1995.tb07041.x PMC398126

[30] Davis C. M. Bustamante P. L. Loach P. A. (1995) Reconstitution of the bacterial core light-harvesting complexes of *Rhodobacter sphaeroides* and *Rhodospirillum rubrum* with isolated α- and β-polypeptides, bacteriochlorophyll *a*, and carotenoid. J. Biol. Chem. 270, 5793–5804 7890709 10.1074/jbc.270.11.5793

[31] Davis C. M. Parkes-Loach P. S. Cook C. K. Meadows K. A. Bandilla M. Scheer H. Loach P. A. (1996) Comparison of the structural requirements for bacteriochlorophyll binding in the core light-harvesting complexes of *Rhodospirillum rubrum* and *Rhodobacter sphaeroides* using reconstitution methodology with bacteriochlorophyll analogs. Biochemistry 35, 3072–3084 8608148 10.1021/bi951777l

[32] Loach P. A. Parkes-Loach P. S. Davis C. M. Heller B. A. (1994) Probing protein structural requirements for formation of the core light-harvesting complex of photosynthetic bacteria using hybrid reconstitution methodology. Photosyn. Res. 40, 231–245 24309942 10.1007/BF00034773

[33] Meadows K. A. Parkes-Loach P. S. Kehoe J. W. Loach P. A. (1998) Reconstitution of core light-harvesting complexes of photosynthetic bacteria using chemically synthesized polypeptides: 1. minimal requirements for subunit formation. Biochemistry 37, 3411–3417 9521662 10.1021/bi972269+

[34] Bustamante P. L. Loach P. A. (1994) Reconstitution of a functional photosynthetic receptor complex with isolated subunits of core light-harvesting complex and reaction centers. Biochemistry 33, 13329–13339 7947741 10.1021/bi00249a020

[35] Liu L. N. Duquesne K. Oesterhelt F. Sturgis J. N. Scheuring S. (2011) Forces guiding assembly of light-harvesting complex 2 in native membranes. Proc. Natl. Acad. Sci. U.S.A. 108, 9455–9459 21606335 10.1073/pnas.1004205108 PMC3111327

[36] de Rivoyre M. Ginet N. Bouyer P. Lavergne J. (2010) Excitation transfer connectivity in different purple bacteria: a theoretical and experimental study. Biochim. Biophys. Acta 1797, 1780–1794 20655292 10.1016/j.bbabio.2010.07.011

[37] Ng I. W. Adams P. G. Mothersole D. J. Vasilev C. Martin E. C. Lang H. P. Tucker J. D. Hunter C. N. (2011) Carotenoids are essential for normal levels of dimerisation of the RC-LH1-PufX core complex of *Rhodobacter sphaeroides*: characterisation of R-26 as a *crtB* (phytoene synthase) mutant. Biochim. Biophys. Acta 1807, 1056–1063 21651888 10.1016/j.bbabio.2011.05.020

[38] McGlynn P. Westerhuis W. H. Jones M. R. Hunter C. N. (1996) Consequences for the organisation of reaction center-light harvesting antenna 1 (LH1) core complexes of *Rhodobacter sphaeroides* arising from deletion of amino acid residues at the C terminus of the LH1 α polypeptide. J. Biol. Chem. 271, 3285–3292 8621732 10.1074/jbc.271.6.3285

[39] Francia F. Wang J. Zischka H. Venturoli G. Oesterhelt D. (2002) Role of the N- and C-terminal regions of the PufX protein in the structural organization of the photosynthetic core complex of *Rhodobacter sphaeroides*. Eur. J. Biochem. 269, 1877–1885 11952789 10.1046/j.1432-1033.2002.02834.x

[40] Ratcliffe E. C. Tunnicliffe R. B. Ng I. W. Adams P. G. Qian P. Holden-Dye K. Jones M. R. Williamson M. P. Hunter C. N. (2011) Experimental evidence that the membrane-spanning helix if PufX adopts a bent conformation that facilitates dimerisation of the *Rhodobacter sphaeroides* RC-LH1 complex through N-terminal interactions. Biochim. Biophys. Acta 1807, 95–107 20937243 10.1016/j.bbabio.2010.10.003

[41] Addlesee H. A. Fiedor L. Hunter C. N. (2000) Physical mapping of *bchG*, *orf427*, and *orf177* in the photosynthesis gene cluster of *Rhodobacter sphaeroides*: functional assignment of the bacteriochlorophyll synthetase gene. J. Bacteriol. 182, 3175–3182 10809697 10.1128/jb.182.11.3175-3182.2000 PMC94504

[42] Young C. S. Beatty J. T. (1998) Topological model of the *Rhodobacter capsulatus* light-harvesting complex I assembly protein LhaA (previously known as ORF1696). J. Bacteriol. 180, 4742–4745 9721320 10.1128/jb.180.17.4742-4745.1998 PMC107492

[43] Mothersole D. J. (2013) Assembly, structure and organisation of photosynthetic membranes, Ph. D. Thesis The University of Sheffield, Sheffield, UK

